# Peer Review of “Beyond Expected Patterns in Insulin Needs of People With Type 1 Diabetes: Temporal Analysis of Automated Insulin Delivery Data”

**DOI:** 10.2196/66595

**Published:** 2024-11-27

**Authors:** Anonymous

**Keywords:** multivariate time series, k-means, clustering, machine learning, temporal patterns, data-driven, OpenAPS, open dataset, type 1 diabetes, insulin needs

*This is a peer-review report for “Beyond Expected Patterns in Insulin Needs of People With Type 1 Diabetes: Temporal Analysis of Automated Insulin Delivery Data.”*

## Round 1 Review

The Word file containing the manuscript [1] is encoded with a mess and not readable.

## Round 2 Review

This paper presents a comprehensive analysis of insulin needs in people with type 1 diabetes (T1D) using automated insulin delivery data. The study aims to uncover unexpected temporal patterns in changes in insulin needs, which could potentially offer new insights into T1D understanding and treatment. The research design and methodology are well-structured and makes use of a wide range of statistical and machine learning tools.

There are a few areas that could be improved or clarified, below are my comments:

1. The study is based on data from 29 individuals. What is the generalizability of the results and conclusions drawn from the analysis? How did the authors ensure the statistical power of the study and make sure the findings are applicable to other cohorts?
2. The paper primarily focuses on identifying patterns in the insulin needs of people with T1D. However, it doesn’t clearly outline how these patterns could be used to predict future insulin needs. A predictive validation of the identified patterns could strengthen the study.
3. Too many unnecessary bullet points and bold text in the paper, which significantly hinders easy reading and understanding.
4. The study acknowledges that factors beyond carbohydrates might influence insulin needs, but it doesn’t delve into what these factors might be. There could be confounding factors such as physical activity, stress, illness, etc, which might have influenced the insulin needs of the participants. The author needs to provide analysis or discussion of these factors.
5. Factors such as age, sex, or duration of diabetes can very likely influence insulin needs. Can the authors add some additional analysis around these factors? I suspect this could provide additional insights and potentially reveal more patterns.
6. It would be beneficial if the authors compared their approach with at least one existing method for analyzing insulin needs in patients with T1D. This would allow for a better understanding of the advantages and limitations of their method.
7. I suggest a more in-depth discussion on the findings, especially focus on how it could be practically applied in the management and treatment of T1D. For instance, how could these patterns help in developing more effective automated insulin delivery systems or in informing patient education and self-management strategies?
8. The study’s reliance on self-reported data might introduce bias or inaccuracies, as this data is subject to memory recall and honesty of the participants. I suggest stating this limitation in the Discussion.

## Abbreviations

T1D: type 1 diabetes
